# Psychosocial correlates in patterns of adolescent emotional eating and dietary consumption

**DOI:** 10.1371/journal.pone.0285446

**Published:** 2023-05-24

**Authors:** Patrece L. Joseph, Carolina Gonçalves, Sasha A. Fleary

**Affiliations:** 1 Gillings School of Global Public Health, University of North Carolina at Chapel Hill, Chapel Hill, NC, United States of America; 2 Department of Child Study and Human Development, Tufts University, Medford, MA, United States of America; 3 Graduate School of Public Health and Health Policy, City University of New York, New York, NY, United States of America; Universitat Politecnica de Catalunya - Barcelona Tech, SPAIN

## Abstract

Unhealthy eating behaviors, such as consumption of unhealthy diet and emotional eating, are common in adolescence and tend to co-occur. However, how these behaviors are patterned may vary among adolescents. This study identified patterns of dietary consumption and emotional eating behaviors in adolescents and examined the sociodemographic and psychosocial (e.g., self-efficacy beliefs and motivation) covariates associated with these eating patterns. Data were from the Family Life, Activity, Sun, Health and Eating study. Latent class analysis was used to estimate adolescent dietary patterns from dietary consumption (i.e., fruits, vegetables, sugar-sweetened beverages, junk food, etc.) and emotional eating variables (i.e., eating when feeling sad or anxious). The sample included 1,568 adolescents (Mean age *=* 14.48-years-old, 49% girls, 55% White). A four-class solution best fit the data (e.g., Bayesian Information Criteria [BIC] = 12263.568, three-class model BIC = 12271.622). Four unhealthy eating behavior patterns were identified: poor diet/high emotional eating, mixed diet/high emotional eating, poor diet/low emotional eating, and mixed diet/low emotional eating. Compared to the poor diet/high emotional eating group, the other groups were less likely to include older adolescents, girls, and adolescents who experienced food insecurity, and more likely to have higher self-efficacy for eating fruits and vegetables and limiting junk foods as well as motivation for consuming fruits and vegetables and limiting junk foods. Our findings highlight adolescents’ complex dietary patterns that include dietary consumption and emotional eating behaviors. Future studies should examine other potential dietary patterns that include emotional eating. Efforts to address unhealthy patterns of adolescents’ dietary consumption and emotional eating behaviors should be expanded.

## Introduction

The consumption of sugar-sweetened beverages, junk foods, or energy-dense foods, and using food for coping are common in adolescence. Estimates suggest that 36% to 63% of adolescents consume sugar-sweetened beverages, junk, and fast foods on a given day [1,2]. Approximately 43% and 16.5% of adolescent girls and boys, respectively, report emotional eating behaviors [3,4] or “eating in response to negative emotions” [5,6]. Sugar-sweetened beverages, junk, and fast-food consumption and emotional eating are associated with depression and greater body mass index [7,8]. Yet, only one longitudinal study has linked emotional eating to adiposy—not measured using body mass index [9,10]. Because adolescent eating behaviors predict adult behaviors and chronic disease risk [11,12], it is critical to investigate the co-occurrence of these eating behaviors among adolescents.

The multiple health behavior framework suggests that risk behaviors co-occur and should be investigated and intervened upon concurrently [13]. Nguyen-Michel [14] found that adolescents who reported emotional eating were more likely to consume sweet, salty, energy dense, and high calorie foods. Multiple studies replicate these findings in other adolescent and adult populations [7,15–17]. However, a few studies show no relationship between diet and emotional eating [18,19]. Konttinen et al. [16] argue that the operationalization of diet as energy and macronutrient intake (i.e., fat and carbohydrates) may account for these null findings. Further, there may be different patterns of co-occurring dietary consumption and emotional eating behaviors, particularly among adolescents. Examining these patterns and their associated factors is important to developing treatment and prevention efforts to address adolescents’ eating behaviors.

Several factors are associated with adolescents’ eating behaviors. In adolescence, major physical and biological changes (i.e., puberty and brain development) require greater nutritional demands on the body [20], while increased autonomy and independence allow youth to take greater responsibility and be more engaged in decision-making for their dietary behaviors [21]. However, adolescents’ dietary decision-making is complicated by their access to food [22,23], knowledge [24], and attitudes and beliefs about food [23,25]. For example, Social Cognitive Theory [26] suggests that self-efficacy beliefs, one’s confidence in their ability to carry out a behavior, is associated with engagement in behavior. Among adolescents, self-efficacy for consuming fruits and vegetables is positively associated with fruit and vegetable intake [25,27], whereas self-efficacy for reducing energy-dense snack intake is negatively associated with snack intake [25]. Self-Determination Theory [28] suggests that individuals are motivated to engage in behaviors that fulfill a psychological need of the self (i.e., competence, autonomy, or relatedness). Motivation is linked to low fast-food consumption [29] and high fruits and vegetables intake among adolescents [30,31]. Research examining associations between these psychosocial variables and emotional eating is limited—though findings suggest that eating self-efficacy is inversely associated with loss of control while eating [32] and motivation is unrelated to disordered eating behaviors in adolescents [33].

### Current study

Previous research has established associations between dietary consumption and emotional eating behaviors [7,15–17]. However, these studies focused on unhealthy dietary consumption (e.g., intake of sweet and salty foods) and did not consider eating behavior patterns. This study used latent class analysis (LCA) to examine patterns of dietary consumption and emotional eating behaviors among adolescents. Previous research found gender differences in dietary behaviors: adolescent boys eat more energy-dense snacks than girls [25] and more girls report emotional eating than boys [8,19,34]. Yet, little information about sociodemographic differences (beyond sex) in adolescents’ emotional eating behaviors exist, though extant research links food insecurity to poor diet in adolescents [35] and emotional eating in young adults [36]. Further, overlap between psychosocial factors associated with adolescents’ dietary consumption and emotional eating behaviors exist. For example, motivation is positively associated with healthy dietary consumption [30,31] and addressed in emotional eating interventions [37]. Therefore, this study also examined the sociodemographic covariates and psychosocial factors associated with adolescents’ eating behavior patterns. The research questions for this study were: (1) what are the combined dietary patterns of emotional eating behaviors and dietary consumption among adolescents? and (2) when compared to the group with the worse dietary patterns, what demographic characteristics are associated with each dietary pattern? The second research question may help identify groups at high risk for unhealthy eating patterns and the potential variables that can be targeted to address these patterns.

## Method

### Procedures

Data were from the Family Life, Activity, Sun, Health and Eating study (FLASHE), developed by the National Cancer Institute to examine psychosocial, generational, and environmental correlates of cancer prevention behaviors in a national sample of parent-adolescent dyads. Institutional review board approval is not required by National Cancer Institute to access FLASHE study data. Data were collected from April 1^st^ to October 6^th^, 2014. Dyads were recruited via print and internet banner advertisement and panelist referrals through Ipsos Consumer Opinion Panel. The sample was matched on key demographics of the United States population (e.g., gender, household income, and race/ethnicity). Potential dyads (N = 5,027) were invited to participate in the study and screened for eligibility: parent over 18-years-old who lived with at least one child, 12-17-years-old, at least half of the time. Information was collected for all adolescents in the household. One adolescent per household was randomly selected to participate in the study until quotas were reached for each age range (i.e., 12–13 years-old, 14–15 years-old, and 16–17 years-old). Upon parent consent, adolescents were sent an email to provide their assent. Eligible dyads completed demographics, diet, and physical activity surveys. Approximately, 1,945 parent-adolescent dyads were enrolled in the study. Due to enrollment non-response or lack of study completion, the final FLASHE sample was not representative of the U.S. population as it included predominately female caregivers, a majority White sample, and a sample with high socioeconomic status. Participants received $5-$10 for survey completion. See Oh et. al [38] and Nebeling et al. [39] for the full study methodology and item development.

Data from participants who completed the adolescent and parent diet and demographic surveys were used (n = 1,667). Participants with missing data on sociodemographic covariates and psychosocial variables were excluded from all analyses (n = 99). The final sample included 1,568 adolescents. Age (F = 1.53, *p* = .216), gender (χ^2^ = 0.71, *p* = .400), race/ethnicity (χ^2^ = 6.07, *p* = .108), and food assistance recipient status (χ^2^ = 2.00, *p* = .157) did not significantly differ between adolescents with and without missing data; however, food insecurity status was significantly different (χ^2^ = 8.00, *p* = .005).

### Measures

#### Sociodemographic covariates

Adolescents indicated their age, gender, and race/ethnicity. Age ranged from 12-17-years-old. Response options for sex were male or female. Race/ethnicity was assessed using two questions: are you Hispanic, Latino/a or of Spanish origin and which one or more of the following would you say is your race? The public dataset included one race/ethnicity variable derived from participants’ responses. Options included non-Hispanic White, Hispanic, non-Hispanic Black or African American, and non-Hispanic Other. For this study, non-Hispanic White served as the reference category as this was the largest racial group. Parent surveys were used for food assistance recipient and household food insecurity items. Parents indicated whether they received assistance from the Supplemental Nutrition Assistance Program, Women, Infants and Children, Temporary Assistance for Needy Families, or Supplemental Security Income. Household food insecurity was assessed using Hager et al.’s [40] to two-item screener for identifying families at risk for food insecurity. Parents indicated how often in the past 12 months (1) they *worried about whether food would run out before getting money to buy more* and (2) *the food they bought did not last and they did not have money to get more*. Response options were dichotomized into food secure (never true for both items) and food insecure (sometimes or often true for one item).

#### Dietary consumption

The FLASHE dietary screener was created for the study [39]. Adolescents indicated how many times they consumed fruits, vegetables (green salad, non-fried potatoes, other vegetables, and beans), sugar-sweetened beverages (sports drinks, energy drinks, soda, sweetened fruit drinks and teas—not counting 100% fruit juice), and junk food (chips, cake, candy, frozen desserts like frozen yogurt, ice cream, etc.) over the past 7 days on a 6-point scale. Response options were recoded into per day consumption according to the National Youth Physical Activity and Nutrition Study [41] as follows: I did not eat or drink the item = 0/day, 1–3 times per week = .286/day, 4–6 times per week = .714/day, 1 time per day = 1/day, 2 times per day = 2/day, and 3 or more times = 3/day. Dietary variables were recoded based on the 2015–2020 Dietary Guidelines for Americans [42]. Fruits and vegetables variables were dichotomized into <2 servings/day and > = 2 servings/day. Recommendations include limited intake of sugar-sweetened beverages and added sugar intake less than 10% of total daily calories, hence sugar-sweetened beverages was dichotomized into < 1 servings/day and ≥ 1 servings/day. Junk food was dichotomized into = < 1 servings/day and > 1 servings/day due to (1) the high frequency of daily junk food consumption in our sample (97.6% of adolescents consumed > = 1 servings/day) and (2) United States Department of Agriculture guidelines that suggest limiting intake of added sugars, saturated fats, and sodium, which are prominent in junk foods [43]. A fast-food item was created by FLASHE study researchers. Adolescents indicated the number of days in the past 7 days they purchased food from a fast-food restaurant. Response options were 0 = 0 days, 1 = 1 day, 2 = 2 days, 3 = 3 days, 4 = 4 days, 5 = 5 days, 6 = 6 days, and 7 = 7 days. Because fast food is high in sodium, fats, and sugars [44,45] and calorie-dense, fast food was dichotomized into 0 days and > = 1 days.

#### Emotional eating behaviors

The two emotional eating behavior items were from the Eating in the Absence of Hunger Questionnaire [46]. Adolescents indicated how often they start or continue to eat when not hungry because they: *feel sad or depressed* (sad eating) or *feel anxious or nervous* (anxious eating). Response options ranged from 1 (never) to 5 (always) and were dichotomized into 0 (never or rarely) and 1 (sometimes, often, and always). There was significant overlap in sad and anxious eating (χ^2^ = 565.72, *p* < .001) as 27.6% of adolescents reported both sad and anxious eating.

#### Self-efficacy

The two self-efficacy items were from the Self-Determination Theory Perceived Competence Scales [47]. Items were self-efficacy for consuming fruits and vegetables (i.e., *I feel confident in my ability to eat fruits and vegetables every day*) and self-efficacy for limiting junk foods (i.e., *I feel confident in my ability to limit the amount of junk food and sugary drinks I eat and drink*). Response options ranged from 1 (strongly disagree) to 5 (strongly agree) and were recoded to range from 0 to 4.

#### Motivation

Motivation items were from the Self-Determination Theory Self-Regulation Questionnaire [47]. Motivation for consuming fruits and vegetables and motivation for limiting junk foods were calculated using four items each that assessed reasons adolescents would eat fruits and vegetables or limit their consumption of junk foods and sugar-sweetened beverages (e.g., *I would feel bad about myself if I didn’t*). Response options ranged from 1 (strongly disagree) to 5 (strongly agree) and were recoded to range from 0 to 4. Confirmatory factor analyses supported one-factor solutions for motivation for consuming fruits and vegetables (α = 0.69) and motivation for limiting junk foods (α = 0.70). Motivation for consuming fruits and vegetables and motivation for limiting junk foods were calculated as the average of the four fruits and vegetables-related items and the four junk food items, respectively.

### Data analyses

Data were analyzed using SPSS Statistics Version 27 [48] and Mplus Version 7.3 [49]. A series of two to six latent class models, using dietary consumption and emotional eating variables as indicators, were estimated. LCA is a statistical procedure that identifies underlying groupings within a heterogenous population. All models included sample weights. Each model was estimated at least three times with twice the number of the start values and iterations to ensure the maximum likelihood solution was obtained. If the log-likelihood value was not replicated, the number of starts and iterations were increased until the value was replicated. The Akaike information criterion (AIC), Bayesian information criterion (BIC), sample BIC, and entropy were used to determine which model best fit the data. Lower values of AIC, BIC, and sample BIC indicate a better fitting model whereas entropy values closer 1 indicate high class separation [50–52]. Item response probabilities > = .30 and = < .70 were used to distinguish classes [50,53]. Estimates between predictor variables (sociodemographic covariates and psychosocial variables) and latent class membership were computed using the automatic three-step method (R3Step in Mplus) as it accounts for error in latent class membership and prevents predictor variables from affecting the latent class solution [54]. In this method, a latent class model with only the latent class indicators is first estimated. Next, a most likely class variable that accounts for measurement error in class membership is created from the latent class posterior distribution. The model is then re-estimated with covariates to ensure predictor variables do not influence the model. Estimates between predictor variables and latent groups were computed using multinomial regression; the group with the poorest dietary pattern was the reference group.

## Results

Descriptive characteristics of sociodemographic covariates, dietary behaviors, and psychosocial variables are presented in Table 1. The total sample included 1,568 adolescents. The average age of adolescents was 14.48-years-old (*SE* = 0.04), 49% were girls, 55% White, 38% lived in a food insecure household, and 18% received food assistance. Nearly 46% of the adolescents in our sample engaged in any emotional eating behavior; approximately 34% of adolescents reported eating while sad and 40% while anxious.

**Table 1:** Sociodemographic, psychosocial, and eating behaviors of adolescents (N = 1568).

Variable	Unweighted frequency N = 1,568	Weighted percent
**Sociodemographic covariates**		
Age^a^	14.48(0.04)	
Gender		
Boy	776	50.7
Girl	792	49.3
Race		
Black	261	13.7
Hispanic/Latinx	155	16.2
Other race	150	15.3
White	1002	54.7
Food assistance		
Non-recipient	1282	81.7
Recipient	286	18.3
Household food insecurity		
Did not experience	983	62
Experienced	585	38
**Dietary behaviors**		
Fruits		
≥2 servings of fruits	1239	79.7
<2 servings of fruits	319	20.3
*Missing*	10	
Vegetables		
≥2 servings of vegetables	1165	76.1
<2 servings of vegetables	357	23.9
*Missing*	46	
Sugary-sweetened beverages		
0 daily servings	278	18.6
>0 daily servings	1229	81.4
*Missing*	61	
Junk food		
< = 1 serving	560	37.9
>1 serving	958	62.1
*Missing*	50	
Fast food intake		
0 times per week	761	47.8
≥1 time per week	798	52.2
*Missing*	9	
**Emotional eating variables**		
Sad eating		
Never or rarely	1026	65.8
Sometimes, often, or always	540	34.2
*Missing*	2	
Anxious eating		
Never or rarely	936	60.5
Sometimes, often, or always	620	39.5
*Missing*	11	
**Psychosocial correlates**		
Self-efficacy for consuming fruits and vegetables^a^	3.03(0.03)	
Self-efficacy for limiting junk foods^a^	2.58(0.03)	
Motivation for eating fruits and vegetables	2.44(0.02)	
Motivation for limiting junk foods^a^	2.96(0.02)	

^a^Mean (SE).

Model fit statistics for the two through six-class models are presented in Table 2 and the AIC, BIC, and sample BIC are depicted in S1 Fig. According to the AIC, BIC, sample BIC, and entropy, the four-class model was the best fitting model. The item response probabilities and results of the multinomial regression for the 4-class model are presented in Table 3. The classes were defined as poor diet/high emotional eating (31%; high junk and sugar-sweetened beverages intake and low fruits and vegetables intake with emotional eating predominantly anxious), mixed diet/high emotional eating (9%; high sugar-sweetened beverages intake with high emotional eating), poor diet/low emotional eating (43%; high SSB and low fruits and vegetables intake with low emotional eating), and mixed diet/low emotional eating (17%; high fruits and SSB intake with low emotional eating).

**Table 2:** Model fit indices of baseline latent class models.

# of classes	-2 log likelihood	AIC	BIC	Sample BIC	Pearson Chi-Square	Posterior probabilities^a^	Entropy	VLMR
2	-6169.067	12368.135	12448.498	12400.846	644.479 df = 112 *p* < .001	665 (0.42) 903 (0.58)	.791	-6481.598 *p* < .001
3	-6051.201	12148.402	12271.626	12198.560	309.513 df = 104 *p* < .001	345 (0.22) 434 (0.28) 789 (0.50)	.744	-6169.067 *p* < .001
4	-6017.742	12097.484	12263.568	12165.088	191.824 df = 96 *p* < .001	266 (0.17) 679 (0.43) 480 (0.31) 143 (0.09)	.814	-6051.201 *p* < .001
5	-6000.089	12078.177	12287.122	12163.228	162.336 df = 88 *p* < .001	289 (0.18) 284 (0.18) 305 (0.19) 152 (0.10) 538 (0.34)	.699	-6017.742 *p =* 0.166
6	-5985.864	12065.727	12317.532	12168.224	138.980 df = 80 *p* < .000	239 (0.15) 85 (0.05) 172 (0.11) 332 (0.21) 477 (0.30) 263 (0.17)	.661	-6000.089 *p =* 1.00

*Note*. AIC = Akaike information criterion; BIC = Bayesian information criterion; VLMR = Vuong-Lo-Mendell-Rubin Likelihood Ratio Test.

^a^Some percentages do not add up to 100 due to rounding. A figure depicting the model fit statistics is included in the supplementary materials.

**Table 3:** Results of the four-class latent class model with sociodemographic and psychosocial covariates.

	Poor diet/high emotional eating	Mixed diet/high emotional eating		Poor diet/low emotional eating		Mixed diet/low emotional eating	
***Latent class Prevalences***	.*32*	.*07*		.*46*		.*15*	
***Average latent class probabilities***	.*92*	.*95*		.*91*		.*91*	
**Item Response Probabilities** ^**1**^							
Fruits	**0**	**0.72**		**0**		**0.73**	
Vegetables	**0.10**	0.55		**0.10**		0.66	
Sugar-sweetened beverages	**0.86**	**0.81**		**0.83**		**0.71**	
Junk food	**0.70**	0.65		0.58		0.54	
Fast food	0.57	0.43		0.54		0.44	
Eat sad	**0.69**	**0.86**		**0.12**		**0**	
Eat anxious	**1**	**0.84**		**0**		**0.07**	
**Covariates**		OR (95% CI)	*p*	OR (95% CI)	*p*	OR (95% CI)	*p*
Age	Ref	0.92 (0.81, 1.04)	.233	**0.91 (0.85, 0.98)**	**.023**	0.89 (0.80, 0.99)	.062
Girl	Ref	1.04 (0.67, 1.04)	.898	**0.51 (0.40, 0.64)**	**< .001**	**0.43 (0.29, 0.63)**	**< .001**
Black	Ref	0.90 (0.47, 1.71)	.773	1.32 (0.97, 1.81)	.201	1.01 (0.61, 1.68)	.973
Latinx	Ref	0.69 (0.32, 1.47)	.330	1.11 (0.75, 1.64)	.685	1.13 (0.65, 1.98)	.734
Other	Ref	0.77 (0.38, 1.54)	.469	1.15 (0.79, 1.67)	.563	1.00 (0.55, 1.82)	.996
Food assistance	Ref	**0.57 (0.31, 1.05)**	**.041**	0.85 (0.63, 1.17)	.370	0.64 (0.38, 1.08)	.080
Food insecurity	Ref	**0.51 (0.32, 0.51)**	**.001**	**0.61 (0.31, 0.79)**	**< .001**	**0.28 (0.28, 0.64)**	**< .001**
Fruits and vegetables self-efficacy	Ref	**2.19 (1.60, 3.00)**	**.004**	1.06 (0.95, 1.19)	0.381	**4.49 (3.04, 6.63)**	**.001**
Fruits and vegetables motivation	Ref	1.58 (1.07, 2.33)	.120	0.92 (0.76, 1.11)	0.419	**2.20 (1.53, 3.19)**	**.015**
Junk food self-efficacy	Ref	0.97 (0.82, 1.16)	.787	**1.38 (1.24, 1.55))**	**< .001**	**1.42 (1.17, 1.72)**	**.012**
Junk food motivation	Ref	1.55 (1.04, 2.31)	.145	**0.80 (0.66, 0.96)**	**.024**	0.98 (0.72, 1.33)	.0897

*Note*. Bolded are any covariates with *p*-values less than .05.

^1^Item response probabilities are for the presence of behaviors. Probabilities >.7 or < .3 are bolded to indicate a high degree of class homogeneity.

Compared to the poor diet/high emotional eating group, the mixed diet/high emotional eating group was significantly less likely to receive food assistance and experience household food insecurity, and more likely to have higher self-efficacy for consuming fruits and vegetables. The poor diet/low emotional eating group was significantly less likely to be older adolescents, girls, experience household food insecurity, and have higher self-efficacy for limiting junk foods, and more likely to have higher motivation for limiting junk foods than the poor diet/high emotional eating group. Compared to the poor diet/high emotional eating group, the mixed diet/low emotional eating group was significantly less likely to be girls and experience household food insecurity, and more likely to have higher self-efficacy for consuming fruits and vegetables, motivation for consuming fruits and vegetables, and self-efficacy for limiting junk foods.

## Discussion

This study identified patterns of adolescents’ eating behaviors (i.e., dietary consumption and emotional eating) and examined sociodemographic covariates and psychosocial variables (i.e., self-efficacy beliefs and motivation) associated with these eating behavior patterns. We identified four eating behavior patterns: poor diet/high emotional eating, mixed diet/high emotional eating, poor diet/low emotional eating, and mixed diet/low emotional eating. Sociodemographic covariates and psychosocial variables differed between adolescents in the poor diet/high emotional eating group and all other groups. Our findings highlight complex patterns of adolescents’ dietary consumption and emotional eating behaviors and provide insight for future research and interventions to address these patterns.

Our identification of poor and mixed diet groups with high emotional eating behaviors aligns with literature documenting the co-occurrence of emotional eating and the intake of energy-dense and sweet foods [7,14,16,17]. Yet, our findings provide nuance to this co-occurrence as there were two groups that were high in emotional eating but had different dietary consumption patterns. To intervene on patterns of adolescents’ eating behaviors, longitudinal studies that assess both emotional eating and dietary consumption are needed. These studies may provide greater understanding about the interplay between food consumption and mental health and the development of eating behaviors in adolescence, which is important as adolescents are at high risk for anorexia, bulimia, and other disordered eating behaviors [12].

Approximately 46% of adolescents in our sample engaged in any high emotional eating behavior, and 27% of adolescents reported eating while both sad and anxious. The prevalence of emotional eating behaviors differed amongst the high emotional eating groups. Item response probabilities indicate that all adolescents in the poor diet/high emotional eating group ate when anxious and the majority when sad. However, there was a mix of sad and anxious eating in the mixed diet/high emotional eating group. Groups also differed in the types of foods they ate; the poor diet/high emotional eating group had high sugar-sweetened beverage and junk foods intake whereas the mixed diet/high emotional eating group had high fruits and sugar-sweetened beverage intake. Our findings add an adolescent population to extant research wherein different emotions are associated with varied dietary behaviors [17]. Because mood is tied to dietary consumption [17], our findings suggest that mental health interventions should address diet. Although mindful eating practices are included in some mental health interventions [55,56], interventions should also address unhealthy dietary intake as a coping strategy.

Our findings suggest that some adolescent demographic groups may be at high risk for emotional eating. Adolescents in the poor diet/high emotional eating group were more likely to experience household food insecurity than adolescents in other groups. This is not surprising as food insecurity is associated with unhealthy diet [35] and other unhealthy eating behaviors among adolescents, such as binge eating and using laxatives [57,58], and may be a strategy for coping with stress associated with food insecurity [59]. We also found gender differences between the high and low emotional eating groups: adolescent girls were less likely to be in the low emotional eating groups than the poor diet/high emotional eating group. This aligns with previous studies that found emotional eating to be more prevalent in girls [8,19,34]. However, emotions not assessed in our study (e.g., confused mood and boredom) have been associated with emotional eating in adolescent boys and young adult males [60]. We found no differences between groups by race/ethnicity and there is limited research on racial and ethnic differences in emotional eating behaviors. Together, these findings suggest that emotional eating behaviors should be included in surveillance studies of adolescent health to identify demographic groups at high risk for emotional eating. This research may provide more information about sociodemographic correlates of emotional eating and facilitate the development of targeted intervention and prevention strategies.

Psychosocial variables also differed among groups. Self-efficacy for consuming fruits and vegetables was higher in the mixed diet groups than the poor diet/low emotional eating group, which aligns with research linking self-efficacy for consuming fruits and vegetables to adolescents’ fruits and vegetables intake [27,61,62]. Future research should examine whether self-efficacy beliefs related to controlling eating behaviors, such as in social and emotional situations, [63,64] may be better predictors of adolescents’ eating behavior patterns. Self-efficacy for limiting junk foods was higher in the low emotional eating groups than the poor diet/high emotional eating group. Emotional eating is tied to high energy-dense and sweet food intake [7,14,16,17] and our results of low emotional eating being related to higher confidence in limiting junk food intake supports this. Compared to the poor diet/high emotional eating group, the mixed diet/low emotional eating group had higher motivation for consuming fruits and vegetables, and the poor diet/low emotional eating group had lower motivation for limiting junk food. Although motivational interviewing is a technique used for initiating behavioral change for fruit, vegetable, and sugar-sweetened beverage intake [65] and targeting emotional eating behaviors in adolescents [37], our findings suggest that this construct may function differently based on adolescents’ eating behavior patterns. Thus, whether adolescents differentially benefit from motivational interviewing techniques based on their dietary patterns should be investigated.

Extant intervention efforts targeting emotional eating may overlook a segment of adolescents who engage in these behaviors. Although many adolescents experience stressors related to increased academic, extracurricular, and social demands [66], some adolescents experience comorbid high stress due to the aforementioned stressors and racial/ethnic discrimination [67], unfavorable environmental conditions (i.e., surplus of fast-food restaurants) [22], and/or limited access to resources (e.g., mental health services)[68]. These adolescents may have fewer strategies for coping with stress and lack support systems for healthy coping when they do have strategies [68]. Given the multiplicative risks encountered by adolescents with comorbid high stress, mental health efforts should address the multiple, intersecting determinants of health related to poor diet, maladaptive coping, and amplified stress. To address these determinants, access to services at a central location with providers who work together to assist the “whole” adolescent through an integrative health care model [69,70] should be expanded. Adolescent-friendly (i.e., private and not requiring caregiver permission) mental health, food, and employment services may be integrated into this multisystem approach, which has demonstrated success [70]. However, to reach adolescents with the greatest need, policies and dedicated funding to support these efforts are essential.

### Limitations

This study has several limitations. The data were cross-sectional; thus, directionality cannot be determined. Future studies should longitudinally assess the relationship between psychosocial variables and adolescents’ eating patterns. The procedures used by the FLASHE team may have resulted in selection bias; however, researchers used several steps to ensure the sample was representative of the U.S. population and included random sampling of adolescents in each household. The dietary screener items used included food groups that were easily categorized as healthy or unhealthy, potentially limiting the ability to discern other types of patterns of adolescent dietary consumption. However, these indicators were selected as they had been associated with adolescents’ emotional eating in previous literature [14,15]. The emotional eating items have not been validated for use as stand-alone items and assessed two emotions, despite research suggesting other emotions, such as happiness and anger [34], may be tied to emotional eating. However, these items are used for emotional eating in published studies using FLASHE data [71,72]. Additionally, the fast-food variable assessed adolescents’ purchase of fast food not fast-food intake, though it is important to note that this variable did not distinguish groups. The psychosocial variables, self-efficacy beliefs and motivation, included combined dietary behaviors (i.e., fruits and vegetables, junk foods, and sugar-sweetened beverages). However, adolescents’ self-efficacy beliefs and motivation likely differ for each dietary behavior (e.g., adolescents may have higher motivation for eating fruits than vegetables). Relatedly, there were no psychosocial variables specific to emotional eating, which may drive dietary consumption among adolescents with high emotional eating behaviors. Despite these limitations, this study provides insight into adolescents’ eating behaviors patterns and psychosocial variables that may be targeted to intervene on these patterns.

### Conclusions

This study identified patterns of adolescents’ eating behaviors and examined associations between these patterns and sociodemographic covariates and psychosocial variables. We identified four patterns of eating behaviors. High emotional eating groups differed in the type of foods consumed and whether sad or anxious eating was prominent. Girls (compared to boys) and adolescents who experienced household food insecurity were more likely to be in the poor diet/high emotional eating group. There were no racial differences between groups. Longitudinal and surveillance studies should include emotional eating behaviors to identify ideal times for intervention and groups at high risk for emotional eating. Further, mental health interventions and integrative health approaches should be expanded to address dietary patterns characterized by emotional eating and unhealthy dietary consumption among adolescents.

## Supporting information

S1 Fig Plot of fit indices for the 2 through 6 latent class models of combined dietary consumption and emotional eating behaviors among adolescents. (TIF)
