# Supplementary figures and images for: Psychosocial correlates in patterns of adolescent emotional eating and dietary consumption
Source: PLoS One. 2023 May 24;18(5):e0285446. doi: 10.1371/journal.pone.0285446 (PMC10208498; doi:10.1371/journal.pone.0285446)

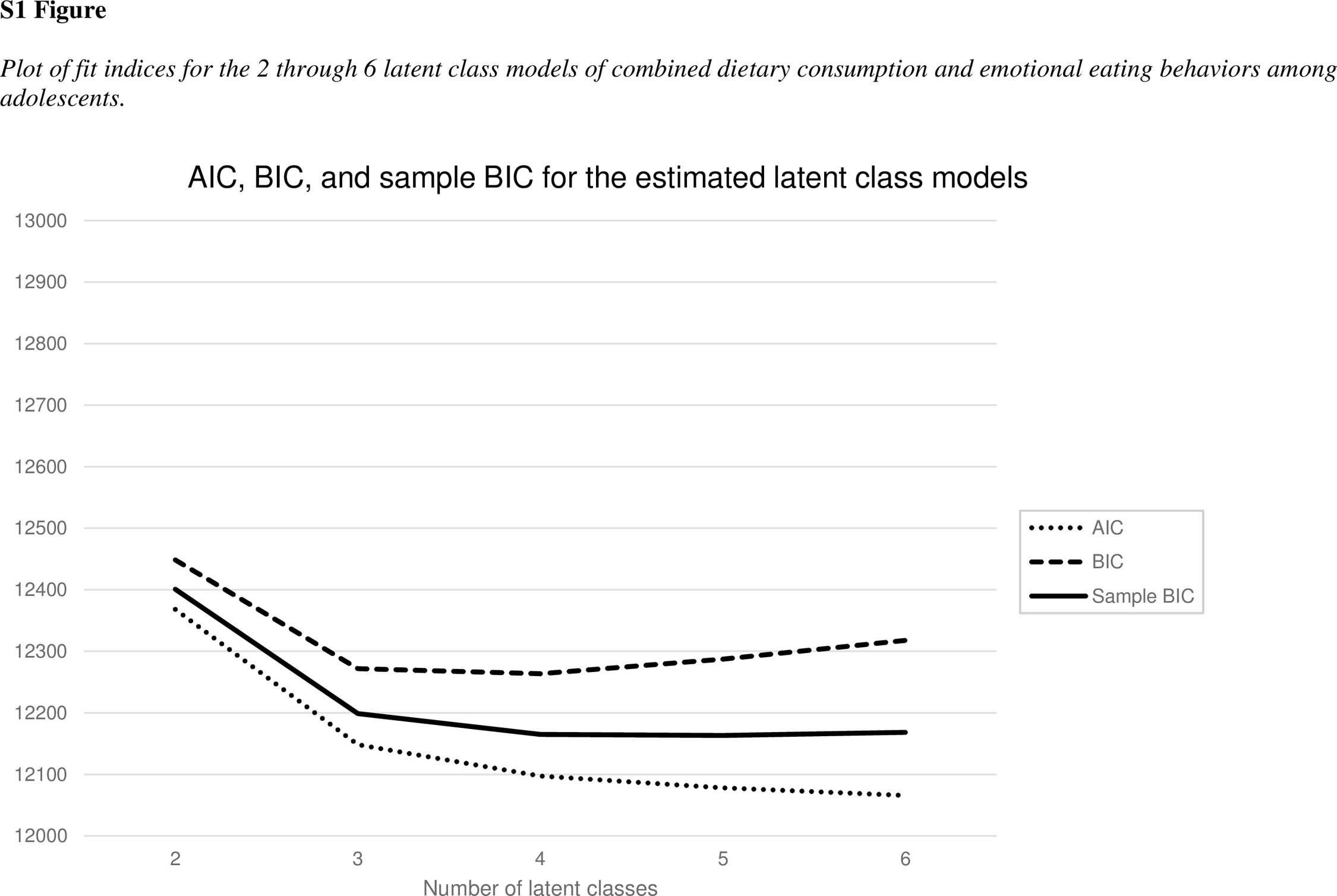

Supplement: S1 Fig — (TIF) [file pone.0285446.s001.tif]
